# Potential of the Non-Contrast-Enhanced Chest CT Radiomics to Distinguish Molecular Subtypes of Breast Cancer: A Retrospective Study

**DOI:** 10.3389/fonc.2022.848726

**Published:** 2022-03-21

**Authors:** Fei Wang, Dandan Wang, Ye Xu, Huijie Jiang, Yang Liu, Jinfeng Zhang

**Affiliations:** ^1^Department of Radiology, Harbin Medical University Cancer Hospital, Harbin, China; ^2^Department of Radiology, The Second Affiliated Hospital of Harbin Medical University, Harbin, China; ^3^Department of Breast Surgery, Harbin Medical University Cancer Hospital, Harbin, China

**Keywords:** breast cancer, molecular subtype, luminal, radiomics, prediction, machine learning

## Abstract

**Objectives:**

The molecular subtype plays an important role in breast cancer, which is the main reference to guide treatment and is closely related to prognosis. The objective of this study was to explore the potential of the non-contrast-enhanced chest CT-based radiomics to predict breast cancer molecular subtypes non-invasively.

**Methods:**

A total of 300 breast cancer patients (153 luminal types and 147 non-luminal types) who underwent routine chest CT examination were included in the study, of which 220 cases belonged to the training set and 80 cases to the time-independent test set. Identification of the molecular subtypes is based on immunohistochemical staining of postoperative tissue samples. The region of interest (ROI) of breast masses was delineated on the continuous slices of CT images. Forty-two models to predict the luminal type of breast cancer were established by the combination of six feature screening methods and seven machine learning classifiers; 5-fold cross-validation (cv) was used for internal validation. Finally, the optimal model was selected for external validation on the independent test set. In addition, we also took advantage of SHapley Additive exPlanations (SHAP) values to make explanations of the machine learning model.

**Results:**

During internal validation, the area under the curve (AUC) values for different models ranged from 0.599 to 0.842, and the accuracy ranged from 0.540 to 0.775. Eventually, the LASSO_SVM combination was selected as the final model, which included 9 radiomics features. The AUC, accuracy, sensitivity, and specificity of the model to distinguish luminal from the non-luminal type were 0.842 [95% CI: 0.728−0.957], 0.773, 0.818, and 0.773 in the training set and 0.757 [95% CI: 0.640–0.866], 0.713, 0.767, and 0.676 in the test set.

**Conclusion:**

The radiomics based on chest CT may provide a new idea for the identification of breast cancer molecular subtypes.

## Introduction

Breast cancer has now overtaken lung cancer to become the highest incidence of cancer in women, with about 2.3 million (11.7%) new cases in 2020; it is the fifth-largest cause of cancer death in the world, with an annual death toll of 685,000 (6.9%) (1). Some commonly used clinical predictors, such as TNM grade and histological grade, cannot fully reflect the heterogeneity of breast cancer (2). In recent years, with the development of molecular biology and sequencing technology, it has been possible to analyze the gene expression profiles of different breast cancer molecular subtypes, using immunohistochemical analysis to further deepen the understanding of the disease at the molecular level (3). There are great differences in the clinical manifestation, treatment response, and prognosis among patients with different subtypes; early identification of molecular subtypes is of great significance for the choice of treatment (4).

According to the expression level of different receptors, the molecular subtypes of breast cancer are composed of luminal A, luminal B, human epidermal growth factor receptor 2 (HER2)-enriched, and triple-negative (TN) type (5). Among them, luminal type (including luminal A and B) is sensitive to endocrine therapy, which is often treated with chemotherapy and endocrine therapy, and the prognosis is good. While the non-luminal type is ineffective to endocrine therapy, the effect of chemotherapy is good, neoadjuvant chemotherapy or targeted therapy and other treatments can be chosen, and the overall prognosis is poor (6). The traditional way to determine the molecular subtypes of breast cancer is usually based on pretreatment biopsy or pathological examination of postoperative tissue samples. But this examination is invasive, time-consuming, and costly, and the limited sample size makes it difficult to fully estimate the heterogeneity within the tumor. Imaging examination is of great importance in the diagnosis of breast cancer. However, traditional imaging examination can only observe the disease from a limited perspective, and it is mostly applied to judge the benign/malignant mass or calcification or assist in preoperative grading, which seriously depends on the experience of radiologists, and it is difficult to describe the lesions from a microscopic point of view (7).

Radiomics can use mathematical methods to quantify the disease information contained in the medical images, and the quantitative value extracted from images can represent the shape, intensity, and texture of tumors; the quantitative value is called a feature, which can be analyzed by different machine learning methods (8). At present, it is not uncommon to use radiomics methods to predict the molecular subtypes of breast cancer, and many encouraging results have been obtained, most of which are based on the radiomics features of breast mammography, ultrasound, or MRI for model development and validation (9–11). CT also plays an important role in the clinical practice of breast cancer (12). Although most of the current guidelines do not recommend the utilization of chest CT as a routine examination for breast cancer diagnosis or early screening (13, 14), in the actual diagnosis and treatment activities, because of the incidence of lung and bone metastasis in breast cancer patients, especially for patients with late clinical stage, chest CT is still one of the routine examinations in most patients. In addition, many breast cancer patients will undergo CT for other reasons (such as chest pain) (15). You et al. explored the prevalence of initial distant metastasis and the benefits of initial chest CT in detecting distant metastasis based on molecular subtypes of breast cancer (16). Song et al. investigated the usefulness of chest CT-based texture analysis to predict overall survival in inflammatory breast cancer patients (17). A prospective study confirmed that the texture and perfusion characteristics of chest CT can effectively predict the expression of histological biomarkers and treatment response in patients with breast cancer (18). Thus, chest CT has great application potential in breast cancer. If we can use the quantitative information from chest CT images to predict the molecular subtypes of patients before treatment, it is very meaningful from the point of view of clinical usefulness and patient economy. In our study, we hypothesized that the tumor quantitative information contained in chest CT can overcome the traditional limitations of qualitative observation by physicians and thus contribute to the identification of the molecular subtype of breast cancer. As far as we know, there is no such research at present.

Therefore, based on the above background, we extracted high-dimensional radiomics features from breast cancer patients’ chest CT images and used this information to establish and validate a machine learning prediction model to recognize the luminal type of breast cancer, which provides new ideas for clinical diagnosis and treatment.

## Materials and Methods

### Patients

**Figure 1:**
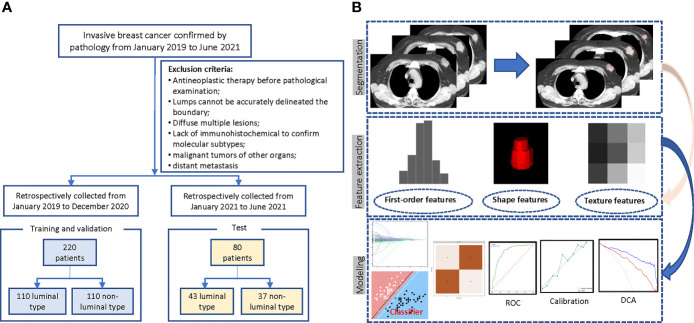
Flowchart of the patient selection and study design. **(A)** Case screening and division of training and test set. **(B)** The radiomics workflow.

A total of 698 female patients with primary breast invasive ductal carcinoma confirmed by pathology and examined by chest CT before treatment in the *Harbin Medical University Cancer Hospital* from January 2019 to June 2021 were retrospectively collected. This study was approved by the Ethics Committee, and because it was a retrospective study, informed consent of the patient was exempted. *Exclusion criteria*: a) antineoplastic therapy before CT (*n* = 186); b) poor image quality, lesion location or boundary difficult to judge, incomplete mass, or artifacts in the image so that a complete ROI sketch could not be performed (*n* = 48); c) diffuse or multiple lesions involving the whole breast (*n* = 23); d) without immunohistochemical examination (*n* = 38); e) combined with malignant tumors of other organs (*n* = 8); and f) distant metastasis before treatment (*n* = 19). In the end, there were 376 eligible patients. To overcome the category imbalance caused by the significantly different incidence of different molecular subtypes, which will affect the fitting effect of the machine learning algorithm, and to avoid selection bias at the same time, we selected 220 consecutive patients composed of the same number of luminal and non-luminal types from January 2019 to December 2020 as the training set, and the rest of the patients during this period were abandoned. In addition, another 80 patients from January to June 2021 were selected as a time-independent test set for external validation. The case screening process is shown in **Figure 1A**.

### CT Image Acquisition Protocol

Non-contrast-enhanced CT scan was performed using spiral CT (GE16, Connecticut Fairfield City, USA) with patients in the supine position. *Scanning parameters*: tube voltage, 100 kV; tube current, 210 mA; 512 × 512 matrix; and slice thickness, 5 mm. *Reconstruction algorithm*: filter type, BODY FILTER; convolution kernel, STANDARD; reconstruction diameter, 390.

### Pathological Assessment

Pathological reports included the expression status of estrogen receptor (ER), progesterone receptor (PR), and HER2. The pathological diagnosis was completed by two pathologists. According to American Society of Clinical Oncology (ASCO) guidelines (5), breast cancer is classified as “luminal A”, “luminal B”, “HER2-enriched”, and “TN”. Samples with positive ER and/or PR expression (more than 1%) were classified as luminal type (including luminal A/B); HER2 enriched type (ER and PR negative, HER2 3+) and triple-negative type (ER, PR, and HER2 negative) were classified as non-luminal type. In addition, HER2 2+ type required fluorescence in situ hybridization (FISH) to further confirmation. Molecular subtypes were labeled independently by a breast surgeon with more than 10 years of experience according to the expression of receptors in the pathological diagnosis report.

### Tumor Segmentation and Feature Extraction

All chest CT images were exported from the picture archiving and communication system (PACS) with DICOM format and converted to Nifty for anonymization. A radiologist with 10 years’ experience in radiology drew the region of interest (ROI) along the boundary of the breast mass manually in the mediastinal window of chest CT, using the open-source platform: the medical imaging interaction toolkit (MITK, https://www.mitk.org/). Radiomics feature extraction was performed by the PyRadiomics package Version2.1.0 (https://pyradiomics.readthedocs.io/) (19). To test the repeatability of manual segmentation, 30 patients were randomly selected. Fourteen days after the first segmentation, the first radiologist and another radiologist with 5 years’ experience performed a secondary segmentation and feature extraction process. The features’ intra-observer and inter-observer intraclass correlation efficient (ICC) were calculated, respectively, and the features with ICC ≥ 0.80 were selected for subsequent analysis. The two radiologists who sketched the ROI knew only that the patient had breast cancer and were blinded to other available clinical and pathological information. For feature extraction, resampledPixelSpacing is [3, 3, 3]. Wavelet, LOG, and LBP3D transform are used to filter the original CT images. Extracted features can be divided into three categories—first order, shape, and texture features—in which texture features include gray co-occurrence matrix (glcm), gray run-length matrix (grlm), gray size region matrix (glzm), gray correlation matrix (gldm), and neighborhood gray difference matrix (ngtdm).

### Machine Learning and Model Performance Evaluation

All features were standardized to a mean value of 0 and SD of 1. To avoid information redundancy and model overfitting caused by high-dimensional features, in the training set, Spearman’s correlation coefficient within each feature is calculated first, and the features’ coefficient greater than 0.90 was deleted. Then, Six feature selection methods, including the least absolute shrinkage and selection operator (LASSO), F test, Pearson’s correlation, mutual information, tree model, and recursive feature elimination (RFE), were used to further reduce the dimension of features and to select the most effective features for predicting luminal type breast cancer.

For the selected features, seven supervised machine learning classifiers were used, including support vector machine (SVM), random forest (RF), extreme gradient boosting (XGBoost), Adaboost, LightGBM, GaussianNB, and multilayer perceptron (MLP), to build radiomics signatures. The 5-fold cross-validation (cv) was used for internal validation to evaluate the robustness and select optimal hyperparameters. In this process, all patients were randomly divided into five groups with the same sample size, four of which were regarded as the initial training set and the rest was the validation set. This process is repeated five times, and the final performance of internal validation was taken as the mean value of the 5-fold cross-validations. A total of 42 models (6 × 7 = 42) were established, and the effectiveness of the model was evaluated in terms of differentiation (AUC, in which closer to 1 means a better model), calibration (calibration curve, which is used to describe the consistency between the predicted results and the real state; the lower the brier score is, the better the predictions are calibrated) and clinical application (decision curve). According to the models’ performance in the internal validation, the hyperparameters with the best model performance were selected, and the whole data (all five groups) were used as the final training set to retrain the model, the model of the optimal combination is selected as the final model, and the independent external validation is carried out in the test set. The above machine learning classifiers are built using the Python3.7 version by the scikit-learn library. The radiomics process is shown in **Figure 1B**. To overcome the “black box” nature of machine learning models and increase the interpretability, we visualized the final model with the SHapley Additive exPlanations (SHAP) dependence plot, which can explain how a single feature affects the output of the LASSO_SVM prediction model. This is a uniform procedure for interpreting the outcome of machine learning models. The SHAP value can be used to estimate the contribution of each feature to the predicted result (20).

### Statistical Analysis

R (V3.6.3, https://www.R-project.org/) and Python (V3.7, https://www.python.org/downloads/) were used for statistical analysis and figure plotting. The Kolmogorov–Smirnov test was used to evaluate the normal distribution of continuous variables. The data with normal distribution and homogeneity of variance were tested by independent sample t-test and expressed by mean [Standard Deviation (SD)]. Otherwise, the data were analyzed by Mann–Whitney U test and expressed as median [interquartile range (IQR)]. The chi-square test was used to compare categorical variables between groups. Two-tailed *p* < 0.05 was defined as statistically significant.

## Results

### Patients

In the total of 300 breast cancer patients (mean age: 61.5 years), 97 (32.3%) belonged to luminal A, 56 (18.6%) to luminal B, 61 (20.3%) to HER2, and 70 (23.3%) to TN. In addition, 16 cases (ER-, PR- and Her2 2+) were categorized as non-luminal type. A total of 132 (44%) patients had positive lymph node metastasis. The median time interval between the patient’s pathological results and the CT images was 23 (IQR: 22–25) days. The detailed clinical and pathological information is shown in **Table 1** and **Supplementary Figure 1**.

**Table 1:** Baseline information of the training and test sets.

Variables	Total (*n* = 300)	Training set (*n* = 220)	Test set (*n* = 80)	*p*-Value
**Age (years)**	61.5 ( ± 10.0)	61.3 ( ± 10.2)	62.2 ( ± 9.2)	0.451
**Diameter (cm)**	2.5 [1.9, 3.0]	2.5 [1.8, 3.1]	2.5 [2.0, 3.0]	0.249
**Ki67 (%)**	25 [15, 40]	25 [15, 40]	30 [15, 40]	0.877
**T stage, *n* (%)**				0.249
T1	77 (25.6)	61 (27.7)	16 (20.0)	
T2	219 (73.0)	157 (71.3)	62 (77.5)	
T3	4 (1.3)	2 (0.9)	2 (2.5)	
**Histological stage, *n* (%)**				0.698
I	7 (2.3)	6 (2.7)	1 (1.3)	
II	180 (60)	130 (59.1)	50 (62.5)	
III	113 (37.7)	84 (38.2)	29 (36.2)	
**Positive lymph nodes, *n* (%)**				0.598
0	168 (56)	122 (55.4)	46 (57.5)	
1~3	80 (26.6)	57 (25.9)	23 (28.7)	
≥4	52 (17.4)	41 (18.6)	11 (13.7)	
**P53, *n* (%)**				0.225
Negative	171 (57)	130 (59.1)	41 (51.3)	
Positive	129 (43)	90 (40.9)	39 (48.7)	
**Molecular subtype, *n* (%)**				0.311
Luminal A	97 (32.3)	69 (31.3)	28 (35)	
Luminal B	56 (18.6)	41 (18.6)	15 (18.7)	
HER2-enriched	61 (20.3)	49 (22.2)	12 (15.1)	
Triple-negative	70 (23.3)	47 (21.3)	23 (28.7)	
Unclear (HER2(2+))	16 (5.3)	14 (6.3)	2 (2.5)	

Data are presented as mean ( ± SD) or median [interquartile range (IQR)] for continuous variables and n (%) for categorical variables.

HER2, human epidermal growth factor receptor 2.

### Machine Learning Model Construction

**Figure 2:**
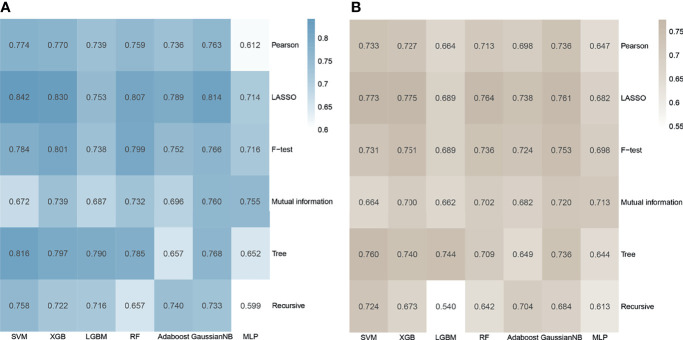
Heatmap of the model performance under different algorithm combinations of feature selection methods (rows) and classification algorithms (columns). **(A)** Area under the curve (AUC) values of the 42 models in the cross-validation. **(B)** Accuracy values of the 42 models.

In the whole cohort, a total of 1561 manual radiomics features were extracted, of which 78 features were removed because their ICC was less than 0.80 (**Supplementary Figure 2**). Then, in the training process, Spearman’s correlation analysis removed 987 highly related features from the training set and left 496 features, of which 112 were first-order features, 4 were shape features, and 380 were texture features. For each feature screening method, no more than 10 features were selected for further modeling to avoid poor performance in the test set caused by overfitting. The AUC values and accuracy of the models under different algorithm combinations are shown in **Figure 2**. The AUC values of the 42 models ranged from 0.599 to 0.842, with the Recursive_MLP model performing the worst and LASSO_SVM performing the best in the internal validation. The accuracy ranged from 0.540 in the Recursive_ LightGBM to 0.775 in the LASSO_ XGBoost model. Therefore, the LASSO algorithm is the most superior as a feature selection method.

In the LASSO algorithm, the result suggested that the number of features is selected between 4 and 26 (**Supplementary Figure 3A**). To ensure the better model effect under the condition of the smaller feature number, the variables in the final model include 9 radiomics features: original_firstorder_10 Percentile, original_glcm_ClusterShade, log-sigma-2-0mm-3D_glcm_Correlation, log-sigma-30-mm-3D_firstorder_90Percentile, log-sigma-5-0-mm-3D_glszm_SmallAreaEmphasis, wavelet-LLH_glszm_LowGrayLevelZoneEmphasis, wavelet-HLL_glszmSmallAreaEmphasis, wavelet-HHH_glcm_InverseVariance, and lbp-3D-m1_firstorder_Maximum (**Supplementary Figure 3B**). **Supplementary Figure 4** shows the ROC, calibration, and decision curves of the seven machine learning models.

### Model Performance Evaluation

**Figure 3:**
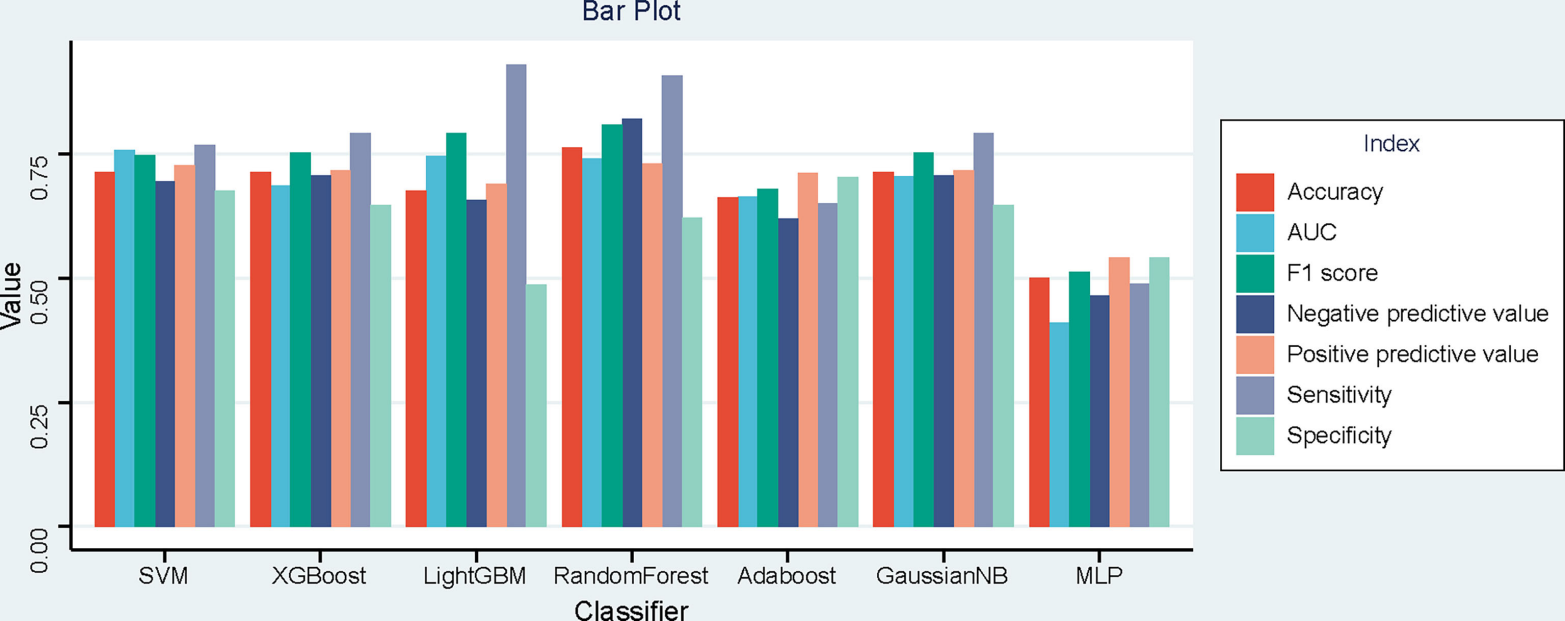
Bar plot of the seven models’ performances in the time-independent external validation based on least absolute shrinkage and selection operator (LASSO).

**Figure 4:**
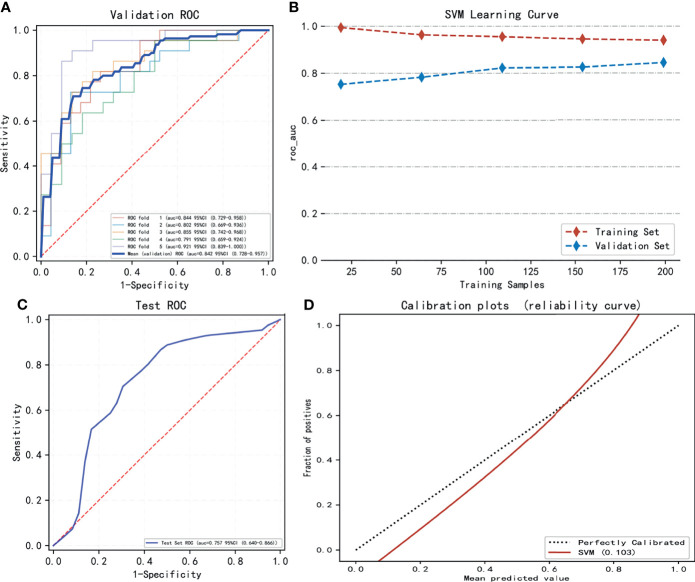
The model training and testing process. **(A)** The area under the curve (AUC) values of LASSO_SVM in internal validation [5-fold cross-validation (cv)]. **(B)** The variation trend of the model’s AUC value with the increasing sample size. **(C)** The AUC value of LASSO_SVM in external validation (test set). **(D)** Model’s calibration curve shows good fitting effect.

**Figure 5:**
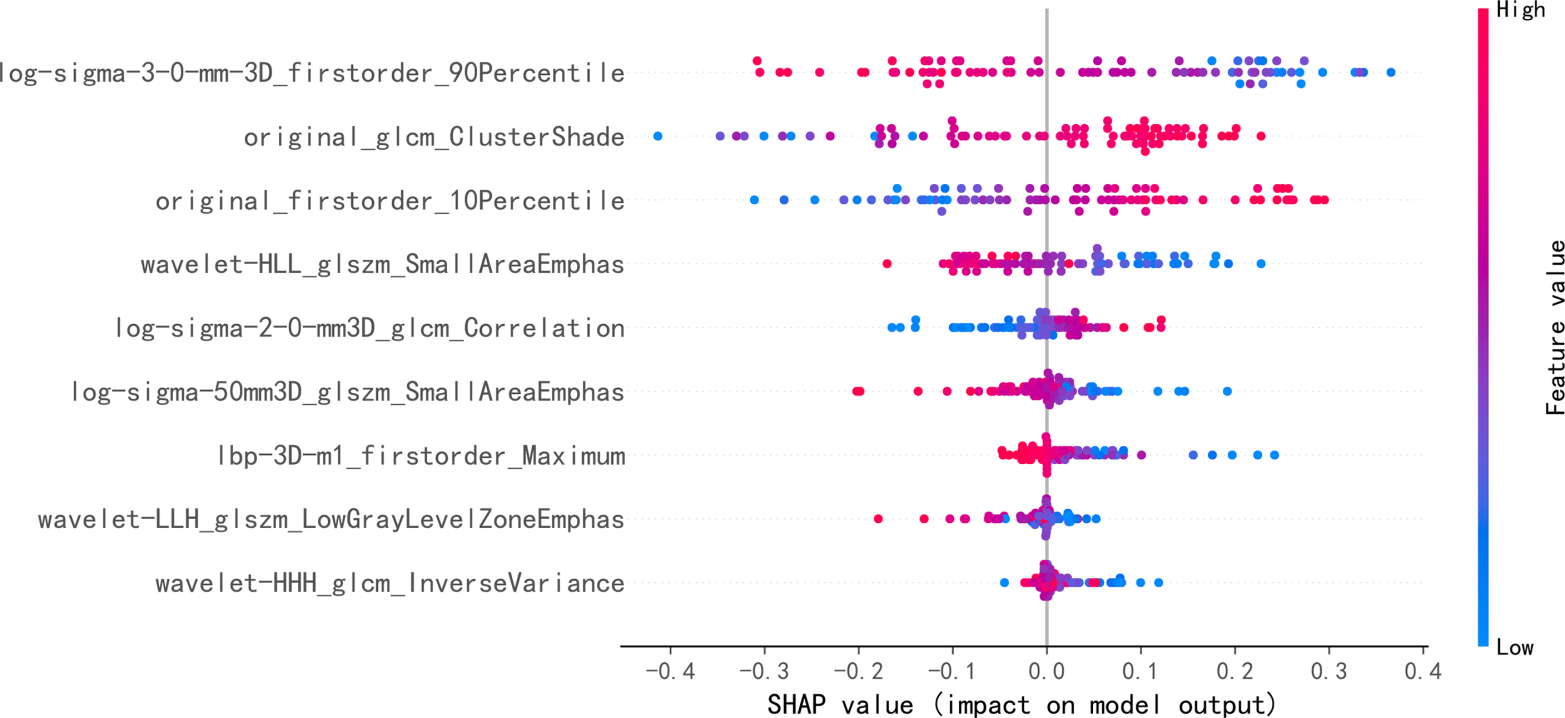
Each point on the graph is a SHapley Additive exPlanations (SHAP) value for a feature and sample. The position on the *y*-axis is determined by the feature, and the position on the *x*-axis is determined by the SHAP value. In this graph, log-sigma-3-0-mm-3D_firstorder_90Percentile, as the most important feature, has a high SHAP value. The color represents the value of the feature from low to high. Overlapping points jitter along the *y*-axis, so we get the distribution of SHAP values for each feature. The features are listed in order of their importance.

The performance of seven machine learning classification models based on the LASSO algorithm in external validation is shown in **Figure 3**: SVM has the highest AUC value (0.757), LightGBM has the highest sensitivity (0.93), RF has the highest accuracy (0.763), and Adaboost has the highest specificity (0.703). The hyperparameters of SVM were set as follows: regularization factor, C = 1; kernel function, rbf; convergence measure, tol = 0.1. The mean AUC was 0.842 (fold 1–5: 0.791–0.921) in the validation set (**Figure 4A**). The learning curve of SVM is shown in **Figure 4B**: with the increase of training samples, the performance of the model in internal validation tended to be stable. The AUC of the final model in the test set was 0.757, 95% CI: 0.640–0.866 (**Figure 4C**); and the calibration curve showed a good fitting effect (**Figure 4D**, Brier score = 0.103). The model evaluation indexes of the training set and test set are shown in **Table 2**. **Figure 5** is the SHAP plot of the visualization for the prediction model, which described the relationship between the high and low features’ SHAP values of the training set. According to the LASSO_SVM model, a dot is created for each feature value of the model for each patient, so a dot is assigned to each patient on each feature line. The dots were colored according to the feature values of their respective patients and vertically accumulated to depict density. Red indicates high feature values, and blue indicates low. The higher the absolute SHAP value of a feature is, the more likely it is luminal type breast cancer.

**Table 2:** Performance of the LASSO_SVM model in the training and test sets.

	Training set	Test set
**AUC**	0.842 (0.728–0.957)	0.757 (0.640–0.866)
**Accuracy (%)**	0.773 (0.681–0.865)	0.713 (0.614–0.812)
**Sensitivity (%)**	0.818 (0.733–0.903)	0.767 (0.674–0.860)
**Specificity (%)**	0.773 (0.681–0.865)	0.676 (0.573–0.779)
**Positive predictive value (%)**	0.772 (0.680–0.864)	0.727 (0.629–0.825)
**Negative predictive value (%)**	0.781 (0.690–0.872)	0.694 (0.593–0.795)
**F1 score**	0.792 (0.703–0.881)	0.747 (0.652–0.842)

The range of values in parentheses indicates the 95% CI.

AUC, area under the curve.

**Figure 6:**
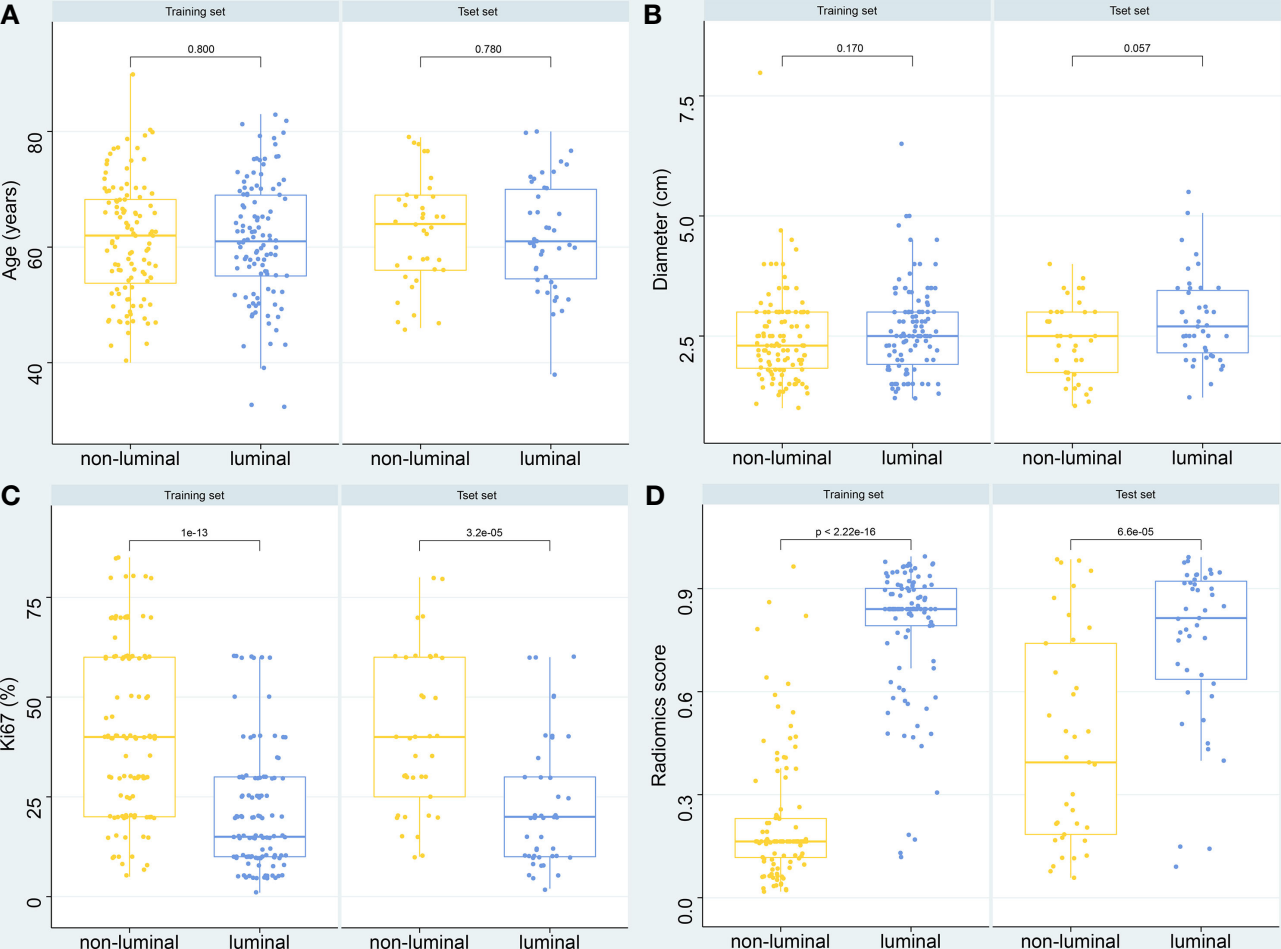
Boxplot of the relationships between different variables and molecular subtypes. **(A)** Age. **(B)** Diameters of breast mass. **(C)** Ki-67 expression level. **(D)** Radiomics score of the LASSO_SVM model.

The predictive probability of each patient belonging to luminal type based on the SVM model was used as a radiomics score. The boxplot (**Figure 6**) further showed the differences in age, tumor diameter, Ki67 expression level, and SVM radiomics scores between luminal and non-normal patients. Among them, Ki67 and radiomics scores were statistically different. **Supplementary Figure 5** shows four typical cases that correspond to the labeled and model predicted molecular subtype classification of the LASSO _SVM model.

## Discussion

Radiomics is a new technology in recent years. In this retrospective study, we constructed a diagnostic model based on the LASSO_SVM classifier containing 9 radiomics features, to explore the application potential in distinguishing luminal from non-luminal breast cancer.

A newly published meta-analysis has shown that there are currently more than 40 studies concerning the radiomics assessment of molecular subtypes of breast cancer, most of which are based on mammography, ultrasound, and MRI (21). Huang et al. found that the combination of radiomics and machine learning based on multi-parameter MRI provides a promising method for the non-invasive prediction of molecular subtypes and androgen receptor expression of breast cancer. The MLP classifier showed the best performance in discriminating triple-negative breast cancer (TNBC) vs. non-TNBC (AUC, 0.965; accuracy, 92.6%) (22). Choudhery et al. found that MRI radiomics features were associated with different breast cancer molecular subtypes in patients treated with neoadjuvant chemotherapy. Significant differences were found in the median volume, median longest axial tumor diameter, and median longest volumetric diameter among different tumor subtypes (*p* = 0.008, 0.009, and 0.01, respectively) (23). Lee et al. (24) investigated the machine learning approaches based on radiomics to predict the molecular subtypes of breast cancer, using the quantitative features extracted from MRI, including perfusion and texture parameters. The RF model achieved the best performance (AUC = 0.80). Although breast MRI has a very high sensitivity, due to the limitations of a regional medical condition and patients’ economic levels, the popularization rate in China is very low. It has been studied that synthetic mammography from digital breast tomosynthesis radiomics signature could discriminate TN, HER2, and luminal subtypes of breast cancer, which yielded an AUC of 0.838, 0.556, and 0.645, respectively, in the validation cohort (25). Niu et al. have studied the evaluation of molecular subtypes of breast cancer by intra-tumor and peritumoral radiomics based on mammography and MRI and constructed signatures using LASSO regression. The AUC of identifying HER2 breast cancer in the validation set can reach 0.907. According to the results of the study, the peritumoral area can provide some supplementary information for prediction, and compared with MRI, the mammography had a higher AUC for distinguishing luminal A from luminal B subtype breast cancer (26). However, although mammography is very common in breast examination, because of its low spatial resolution and in young patients with rich glands, the display effect of mass is not satisfactory. At present, it is often used to assist in the diagnosis of benign and malignant calcification.

Chest CT, although morphological, so far cannot be used for routine screening of breast masses, but for most patients, it is still one of the routine examinations for admission. It is non-invasive and time-saving does not need a contrast agent, and because of its tomographic characteristics, it also has the potential to provide relatively rich focus information, with the improvement of big data’s analytical ability. It will be beneficial to the full mining of this information and to explore their correlation with the biological characteristics of the disease. Yang et al. (27) used manual and deep radiological features based on multi-detector CT (MDCT) to evaluate the status of HER2 in breast cancer patients. The combined model with handmade and deep radiological signatures showed good discrimination, and the C index in the main cohort reached 0.829. These features can provide supplementary help for radiological assessment of the HER2 status of breast cancer. In another radiomics study based on chest CT images, the LASSO logical method was used to construct a prediction model. The AUC values to distinguish TNBC from non-TNBC were 0.881 and 0.851 in the discovery and validation groups, respectively (28). In our study, we tried 42 different feature screening and machine learning classifiers for modeling to predict luminal breast cancer. The AUC, accuracy, sensitivity, and specificity in the test set are 0.757, 0.713, 0.767, and 0.676, respectively. About feature dimensionality reduction methods, the overall performance of the LASSO algorithm is better than other dimensionality reduction algorithms in terms of AUC and accuracy, whether internal or external validation. LASSO is a kind of compressed estimation, which obtains a more refined model by constructing a penalty function. The basic idea is to minimize the sum of squares of residuals when the sum of absolute values of regression coefficients is less than a certain constraint so that some regression coefficients that are strictly equal to 0 can be produced, and an interpretable model can be obtained. It is a biased estimation for complex collinear data, so it can screen variables and reduce the complexity of the model (29, 30). At present, LASSO is the most widely used dimensionality reduction method that can effectively prevent overfitting in different application scenarios (31–33). In the machine learning classifiers based on LASSO dimensionality reduction, the performances of the models are still different. SVM has the highest AUC value in both internal and external validations. In the internal validation, the accuracy of XGBoost is slightly higher than that of SVM, while in the external validation, the two have the same accuracy. Although LightGBM has the highest sensitivity (0.93) in the external validation, the specificity is relatively low (0.486). SVM is a binary classification model, and the core idea is a hyperplane defined in the feature space, which can maximize the geometric interval between different categories, but at the same time, it can also carry out a variety of kernel transformations. This also makes it an essentially non-linear classifier, which is widely used in a variety of scenarios, especially showing great advantages for biomedical classification problems (34–36). SVM is also widely used in breast cancer research. The accuracy of a breast cancer diagnosis can be improved by using radiomics and SVM of multi-parameter breast MRI (37). Zhang et al. constructed a prediction model based on the LASSO feature selection method and SVM classifier by using multimodal MRI radiomics, which could distinguish benign and malignant breast cancer with an AUC value of 0.836 (38). In a study of preoperative MRI radiomics in patients with oropharyngeal squamous cell carcinoma, the LightGBM model showed an AUC of 0.8333 in predicting human papillomavirus (HPV) status and 0.857 in predicting disease recurrence (39). The XGBoost algorithm has been used for feature selection and model building to predict axillary lymph node metastasis in breast cancer, achieving an accuracy of 80%, using the F-18 fluorodeoxyglucose PET/CT (40). MLP is a kind of artificial neural network with a forward structure, which maps a set of input vectors to a set of output vectors. It can follow the principle of the human nervous system to learn and predict data (41). According to Yun (42), a robust classification model was constructed by using the radiomics features based on MRI, which can distinguish glioblastoma from primary central nervous system lymphoma. MLP classifier served a high-performing and generalizable model. Mao et al. (43) constructed multi-classifier-based ultrasound radiomics models, which can be used to identify primary and metastatic liver cancer, in which the logistic regression model outperforms MLP (AUC 0.816 *vs.* 0.790). In our study, the performance of MLP is the worst among all performance indicators, and the possible reason may be that the final effect of the algorithm is closely related to the generalization ability of the network and learning samples, which is particularly obvious in the neural network. If the sample set is poorly representative, there are many contradictory or redundant samples, and it is difficult for the network to achieve the expected performance (44).

As the first exploratory research, our study inevitably has limitations. First of all, as a research on the development and validation of predictive models, although time-independent external validation has been carried out, our data came from a single center; and the application value of CT in breast cancer is still controversial, which needs to be verified on a larger scale and in more centers. Secondly, the slice thickness of our images is large, which may lead to the exclusion of small tumors from the study. In the next work, we plan to prospectively collect thin-slice images to verify the applicability of small tumors. Finally, due to the nature of the retrospective study, there is selection bias, which can also be compensated by future prospective validation. Despite the above limitations, our research is still enlightening and has potential.

## Conclusion

This study explored the potential of non-contrast-enhanced CT imaging in predicting the luminal type of breast cancer and achieved encouraging results, which has some implications for clinical work, but further prospective validation and studies combined with other examinations are still needed.

## Data Availability Statement

The raw data supporting the conclusions of this article will be made available by the authors, without undue reservation.

## Ethics Statement

The studies involving human participants were reviewed and approved by Harbin Medical University Cancer Hospital. Written informed consent for participation was not required for this study in accordance with the national legislation and the institutional requirements. Written informed consent was not obtained from the individual(s) for the publication of any potentially identifiable images or data included in this article.

## Author Contributions

FW and DW contributed to conception and design of the study. YL organized the database. FW performed the statistical analysis. DW and YX drew the ROI. DW wrote the first draft of the manuscript. JZ supervised the project. HJ designed and revised the manuscript. All authors approved the submitted version.

## Funding

This work was supported by the National Natural Science Foundation of China (Grant Number 81802649), Ba Jian Qing Nian Grant of Harbin Medical University Cancer Hospital (Grant Number BJQN2019-09), and Haiyan Grant of Harbin Medical University Cancer Hospital (Grant Number JJQN2018-05).

## Conflict of Interest

The authors declare that the research was conducted in the absence of any commercial or financial relationships that could be construed as a potential conflict of interest.

## Publisher’s Note

All claims expressed in this article are solely those of the authors and do not necessarily represent those of their affiliated organizations, or those of the publisher, the editors and the reviewers. Any product that may be evaluated in this article, or claim that may be made by its manufacturer, is not guaranteed or endorsed by the publisher.

## Abbreviations

ICC, intraclass correlation coefficient; LASSO, least absolute shrinkage and selection operator; ROC, receiver operating characteristic; AUC, area under curve; SVM, support vector machine; RF, random forest; SHAP, SHapley Additive exPlanations; XGBoost, extreme gradient boosting; MLP, multilayer perceptron.
