# Supplementary material for: Potential of the Non-Contrast-Enhanced Chest CT Radiomics to Distinguish Molecular Subtypes of Breast Cancer: A Retrospective Study
Source: Front Oncol. 2022 Mar 21;12:848726. doi: 10.3389/fonc.2022.848726 (PMC8979294; doi:10.3389/fonc.2022.848726)
Supplement: Supplementary file 1 [file DataSheet_1.docx]

Supplementary Material


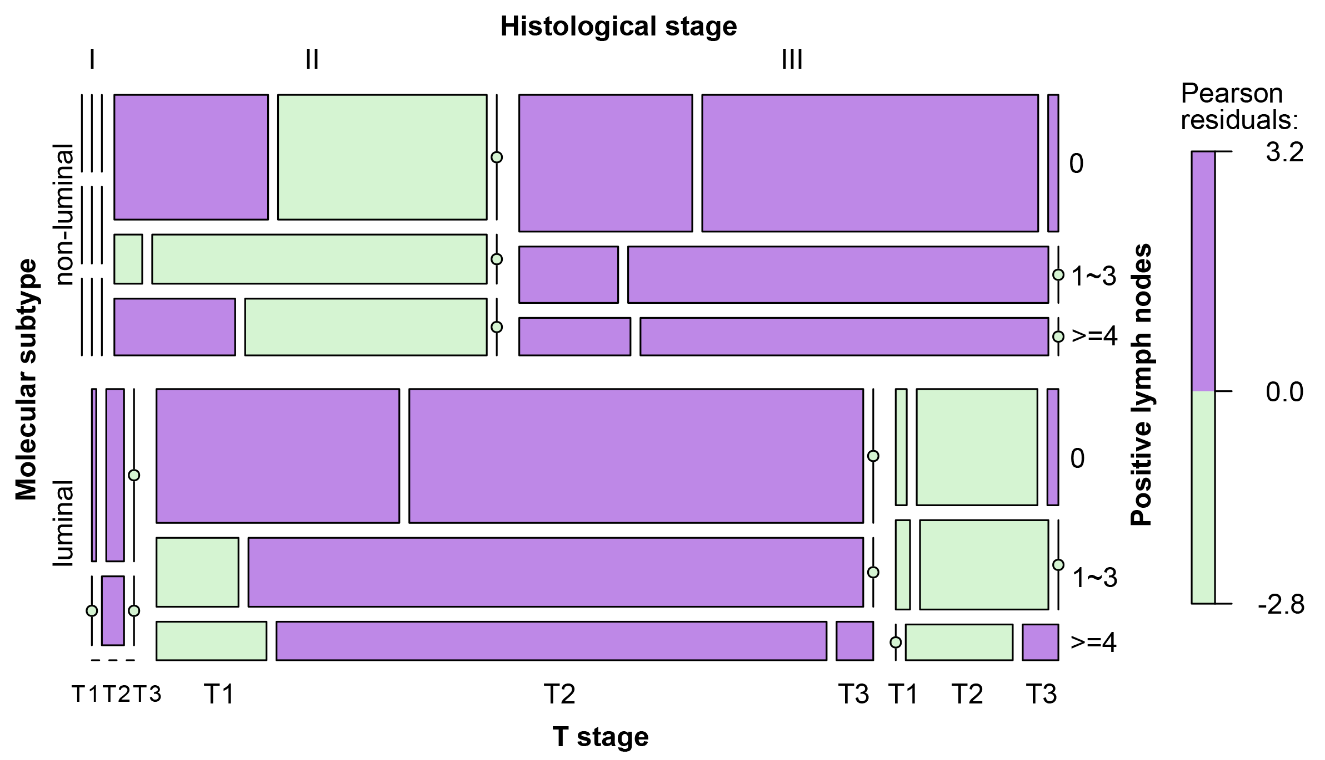


**Supplementary Figure 1.** The schematic of the composition of the category variables in all cohort, and the four directions represent Molecular subtype, Histological stage, Positive lymph nodes number, and T stage respectively.


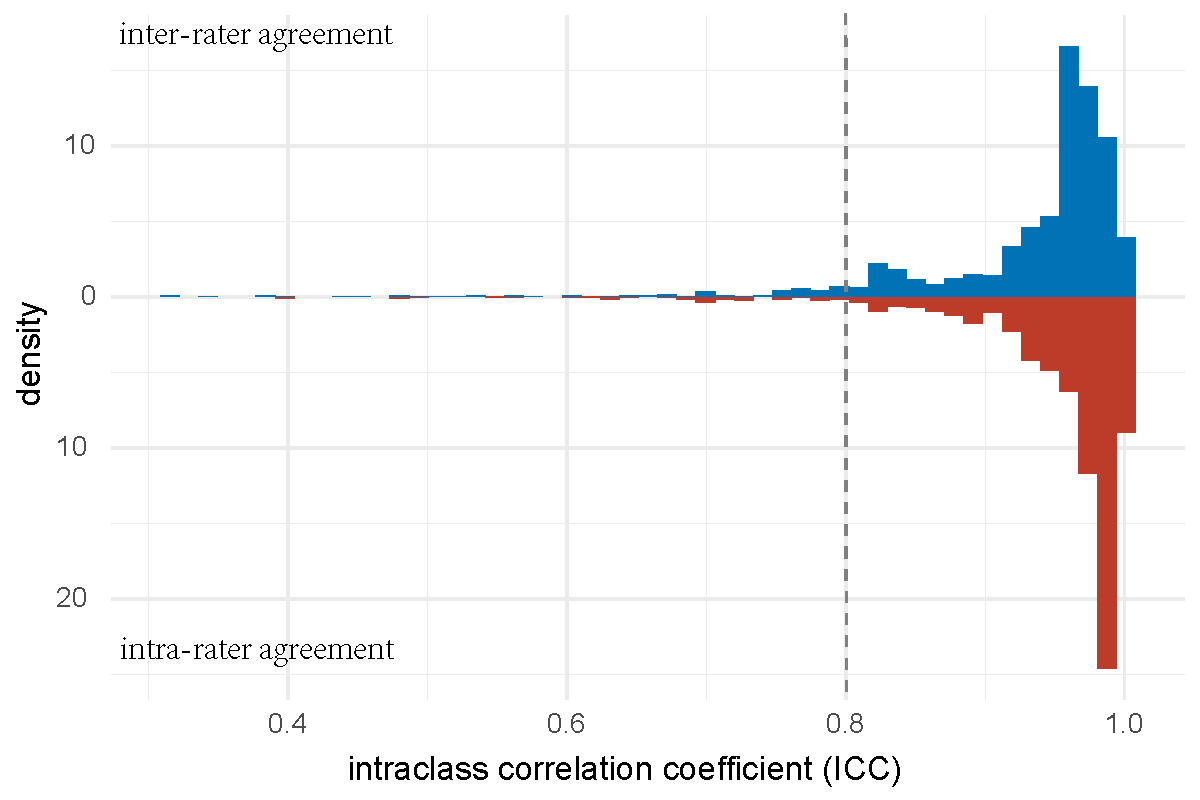


**Supplementary Figure 2.** The bars of intra- and inter-observer’s ICC. Upper: inter-rater agreement; Lower: intra-rater agreement. *ICC: intra-class correlation coefficient.*


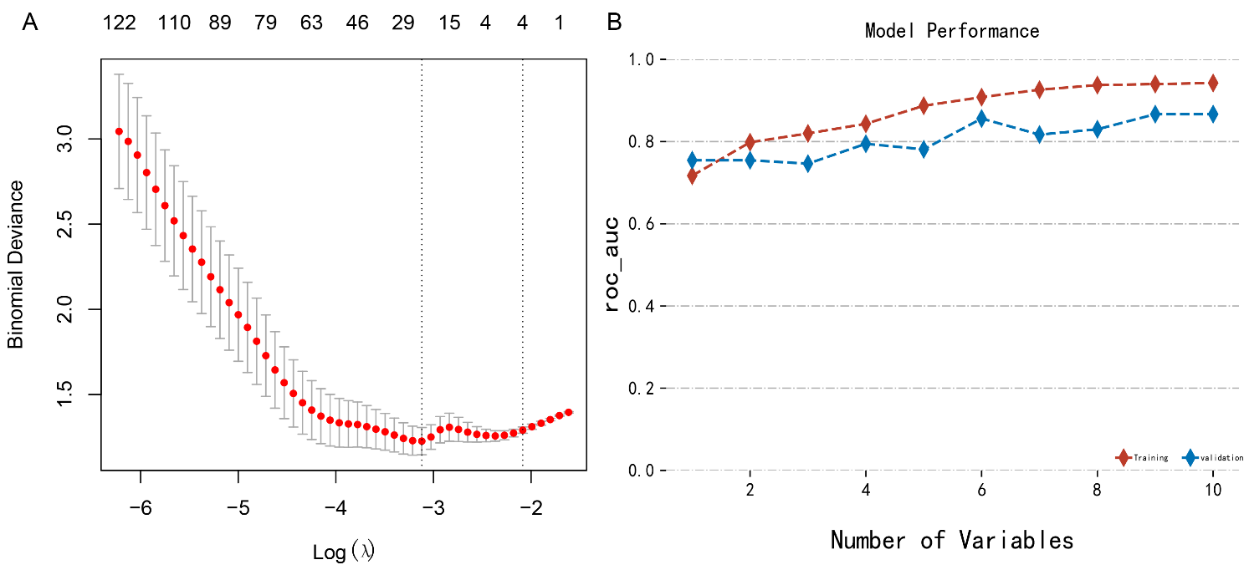


Supplementary Figure 3. Feature number selection for the LASSO_SVM algorithm. (A) cross validation of LASSO: the suggested feature number was between 4 and 26 (λ value ranged from 0.113 to 0.040, and log(λ) ranged from -2.180 to -3.218); (B) AUC values ranged with the feature number during the training and internal validation process.


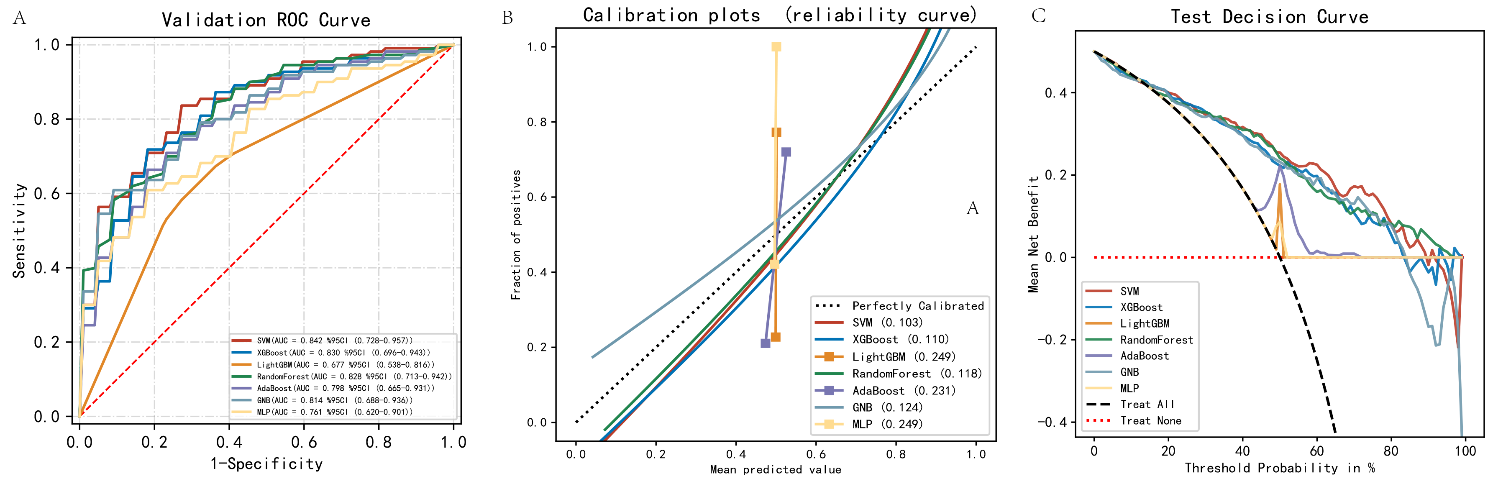


**Supplementary Figure 4.** Performance of selected features based on LASSO algorithm in different machine learning classier. (A) ROC curves of seven machine learning models; (B) calibration curves; (C) decision curves.


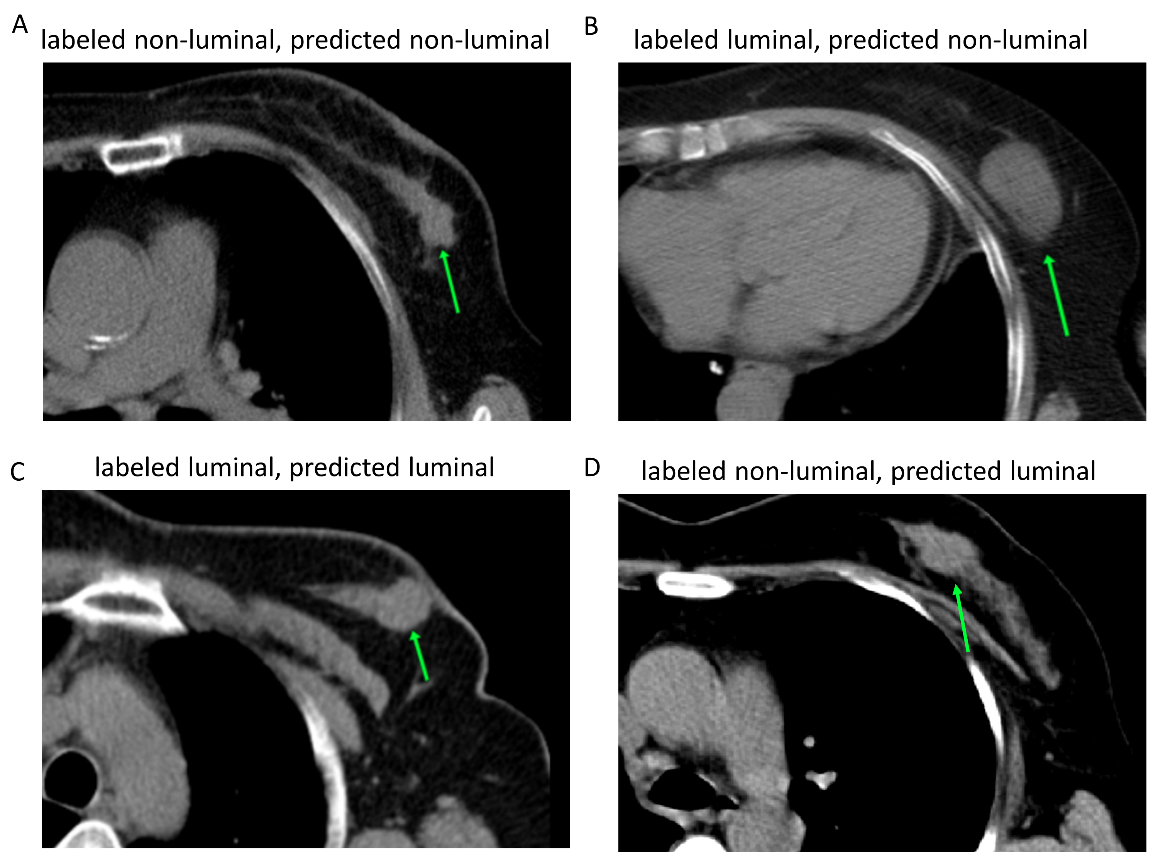


Supplementary Figure 5. The four typical cases correspond to the labeled and model predicted molecular subtype classification respectively.
